# Triptolide-Mediated Apoptosis by Suppression of Focal Adhesion Kinase through Extrinsic and Intrinsic Pathways in Human Melanoma Cells

**DOI:** 10.1155/2013/172548

**Published:** 2013-05-08

**Authors:** Haw-Young Kwon, Kyoung-Sook Kim, Ji-Sue Baik, Hyung-In Moon, Ji-Won Lee, Cheorl-Ho Kim, Young-Su Cho, Yong-Kee Jeong, Young-Choon Lee

**Affiliations:** ^1^College of Natural Resources and Life Science, Dong-A University, Busan 604-714, Republic of Korea; ^2^Medi-Farm Industrialization Research Center, Dong-A University, Busan 604-714, Republic of Korea; ^3^Molecular and Cellular Glycobiology Unit, Department of Biological Sciences, Sungkyunkwan University, Kyunggi-Do 440-746, Republic of Korea

## Abstract

Triptolide (TPL) has been shown to inhibit cell proliferation and induce apoptosis in various human cancer cells; however, the precise mechanism of apoptosis induced by TPL in human melanoma cells has not yet been elucidated. In this study, we investigated the precise mechanism underlying cytocidal effects of TPL on human melanoma cells. Treatment of human melanoma cells with TPL significantly inhibited cell growth and induced apoptosis, as evidenced by flow cytometry and annexin V-fluorescein isothiocyanate analyses. TPL increased the levels of Fas and Fas-associated death domain (FADD) and induced cleavage of Bid by activation of caspase-8 and cytochrome c release from mitochondria to the cytosol, which resulted in activation of caspase-9 and caspase-3. Moreover, TPL-induced apoptosis in SK-MEL-2 cells was mediated through dephosphorylation of focal adhesion kinase (FAK) and its cleavage by caspase-8-mediated caspase-3 activation via upregulation of Fas expression. We also found that TPL mediated the dissociation of receptor-interacting protein (RIP) from FAK and enhanced the formation of RIP/Fas complex formation initiating cell death. In conclusion, our data firstly demonstrated that TPL induces apoptosis by both extrinsic and intrinsic apoptosis pathways in human melanoma cells and identified that RIP shuttles between Fas and FAK to mediate apoptosis.

## 1. Introduction

Malignant melanoma is the most aggressive form of skin cancer that develops from a neoplastic transformation of melanocytes [1]. It presents a significant public health problem as its incidence is increasing faster than that of any other cancer in the USA and has increased at a rate of 3.1% per year over the last two decades [1]. Although melanoma at early stages is effectively curable by surgical treatment, patients with advanced and metastatic melanoma have a very poor prognosis, with approximately 13,000 annual deaths and a median overall survival of 8 to 18 months [2]. Because metastatic melanoma is resistant to a variety of cancer therapies, including combination chemotherapy and immunotherapy, it is important to explore more effective therapeutic agents for treating advanced melanoma.

Apoptosis, which is characterized by specific morphological and biochemical features in which caspase activation plays a key role, is a typical form of cell death that plays an important role in several biological processes such as development, homeostasis, and several diseases [3]. Apoptosis is an important phenomenon in cancer chemotherapy, because a number of cancer cells undergo apoptotic cell death by treatment with chemotherapeutic agents, indicating that apoptosis in cancer cells plays a crucial role in cancer cell killing induced by chemotherapy [4, 5]. However, although chemotherapy is one of the most extensively used anticancer therapies, its effectiveness and safety remain a primary concern due to severe toxicity and significant side effects of chemotherapy [6]. In recent years, a number of natural products isolated from Chinese medicinal herbs have been known to inhibit cell proliferation and angiogenesis, induce apoptotic or nonapoptotic cell death, suppress metastasis, and enhance the anticancer effects of chemotherapeutic agents, which shows anticancer potential both *in vitro* and *in vivo* [6, 7]. As a new source of anticancer drugs, thus, natural compounds and their derivatives exerting their antitumor effect against cancer cells by inducing apoptosis have gradually gained considerable attention in order to reduce chemotherapy-associated side effects.

Triptolide (TPL), a diterpenoid triepoxide, is the major active component of the Chinese medicinal herb *Tripterygium wilfordii* Hook f. that has been used for centuries to treat a variety of autoimmune and inflammatory diseases [8, 9]. In recent years, a number of studies have demonstrated that TPL could inhibit cell proliferation and induce apoptosis in various human tumor cell types including pancreatic cancer [10, 11], gastric cancer [12], myeloma [13, 14], myeloid leukemia [15], glioblastoma [16], colon cancer [17], thyroid carcinoma [18], and adrenal cancer cells [19]. In addition, these studies indicated that TPL not only induces cancer cell apoptosis directly via the mitochondrial (intrinsic) and the death receptor-mediated (extrinsic) pathways, but also enhances apoptosis induced by cytotoxic agents such as TNF-*α*, TRAIL, and chemotherapeutic agents including 5-fluorouracil and dexamethasone [9].

Although TPL-induced apoptosis has been very recently reported in human melanoma A375 cells [20], the precise mechanism by which TPL induce apoptosis in human melanoma cells is not clearly understood. Thus, the aim of this study was to investigate the mechanism underlying TPL-induced apoptosis in human melanoma cells. In this study, we firstly found that TPL induced apoptosis through both mitochondrial (intrinsic) and the death receptor-mediated (extrinsic) pathways in human melanoma SK-MEL-2 cells. Furthermore, present study firstly demonstrated that TPL-induced apoptosis in human melanoma cells was mediated through suppression of focal adhesion kinase (FAK) by caspase-3.

## 2. Materials and Methods

### 2.1. Materials

Triptolide (TPL) was obtained from Sigma Chemical Co. (St. Louis, MO, USA). The antibodies against Fas, Fas ligand (FasL), Fas-associated protein with death domain (FADD), Bid, Bcl-2, Bax, cytochrome c, RIP, and Poly (ADP-ribose) polymerase (PARP) were obtained from Santa Cruz Biotechnology (Santa Cruz, CA, USA); antibodies against caspase-3, caspase-8, caspase-9, Bid, p-FAK, and FAK were purchased from Cell signaling (Beverly, MA, USA); glyceraldehydes-3-phosphate dehydrogenase (GAPDH) was purchased from Millipore Corporation (Temecula, CA, USA). Secondary antibodies, horseradish peroxidase-conjugated goat anti-mouse and rabbit Ig G were purchased from Stressgene Biotechnologies (Victoria, BC, Canada). Propidium iodide (PI) was obtained from Sigma (St. Louis, MO, USA). Annexin V-FITC apoptosis detection kit and Bradford protein assay kit were obtained from BD Biosciences (San Jose, CA, USA) and Bio-Rad Laboratories (Hercules, CA, USA), respectively. Z-IETD-FMK (caspase-8 inhibitor), and Z-LEHD-FMK (caspase-9 inhibitor), Z-VAD-FMK (pan-caspase inhibitor) were purchased from Calbiochem (San Diego, CA, USA). G-agarose bead was obtained from Santa Cruz Biotechnology (Santa Cruz, CA, USA).

### 2.2. Cell Culture

SK-MEL-2 human melanoma, SK-MEL-28 human melanoma, and B16F10 mouse melanoma cell lines were obtained from American Type Culture Collection (Rockville, MD, USA). The cells were grown in Dulbecco's modified Eagle's medium (DMEM; WelGENE Co., Daegu, Republic of Korea) supplemented with 10% (v/v) heat-inactivated fetal bovine serum (FBS), 100 U/mL penicillin, and 100 *μ*g/mL streptomycin at 37°C under 5% CO_2_.

### 2.3. Cell Viability Assay

As described previously [21], cell viability was determined by reduction of 3-(4,5-dimethylthiazol-2-yl)-2,5-diphenyltetrazolium bromide (MTT) to formazan. Briefly, cells were plated in 96-well culture plate (1 × 10^4^ cells/well). After 24 h, the cells were treated with various concentrations of triptolide for the indicated times. After TPL treatment for 24 h, cells were washed with PBS and MTT (0.5 mg/mL) was added to each. After incubation for 4 h, DMSO was added to dissolve the formazan from MTT reduction, and the amount of formazan salt was determined by measuring the OD at 490 nm using an ELISA plate reader (Bio-Rad, Hercules, CA, USA). Cell viability was quantified as a percentage compared to the control.

### 2.4. Flow Cytometric Analysis

To analyze DNA content, cells were treated with various concentrations of TPL for 24 h. After treatment, the cells were collected and fixed with 70% ethanol. Cells were suspended in PBS containing 0.5 mg/mL propidium iodide, 0.5 mg/mL RNase, and 0.03% NP-40, incubated in the dark for 30 min at room temperature, and analyzed using a Beckman-Coulter Cytomics FC500 flow cytometer and CXP software (Beckman-Coulter, Miami, FL, USA). To determine apoptosis, TPL-treated cells were washed in PBS, stained using the Annexin V-FITC Apoptosis Detection Kit according to the instructions of the manufacturer, and analyzed by flow cytometry. Each experiment was repeated at least twice to ensure reproducibility.

### 2.5. Western Blot Analysis

Western blotting was performed as described previously [21]. For total protein preparation, cells were lysed in M-PER buffer (Pierce, Rockford, IL, USA) containing protease and phosphatase inhibitor cocktail. Lysate was centrifuged at 14,000 ×g for 15 min at 4°C.

The protein concentration was measured using the Bradford protein assay kit. Total proteins were separated by electrophoresis, transferred onto a PVDF membrane by electroblotting, and then probed using primary antibodies. The detection of specific proteins was carried out with an enhanced Chemiluminescence Kit (Amersham Biosciences, UK) according to the recommended procedure. Mitochondrial and cytosolic fractions were prepared by Mitochondria Isolation Kit (Pierce, Rockford, IL, USA) according to the instructions of manufacturer. Equal loading was assessed using anti-GAPDH antibody to normalize the amount of total protein. The densitometric intensity of each band was measured with a Scion Image Instrument (Scion Corp., Frederick, MD, USA).

### 2.6. Immunoprecipitation

For total protein preparation, cells were lysed in M-PER buffer (Pierce, Rockford, IL, USA) containing protease and phosphatase inhibitor cocktail. Lysate was centrifuged at 14,000 ×g for 15 min at 4°C. The protein concentration was measured using the Bradford protein assay kit. Also, 500 *μ*g of total protein were incubated with 2 *μ*g of RIP-specific antibody for 2 h followed by incubation with protein G-agarose beads for 2 h at 4°C. Precipitates were washed three times in lysis buffer, and beads were resuspended in 5 × sample loading buffer, while SDS-PAGE was performed with the supernatant.

### 2.7. Reverse Transcription-Polymerase Chain Reaction (RT-PCR)

Total RNA was isolated from SK-MEL-2 cells using Trizol reagent (Invitrogen; Carlsbad, CA, USA). Two micrograms of RNA were subjected to reverse transcription with random nonamers utilizing Takara RNA PCR kit (Takara Bio; Shiga, Japan) according to the manufacturer's protocol. The cDNA was amplified by PCR with the following primers: Fas (365 bp), 5′-TCT AAC TTG GGG TGG CTT TGT CTT C-3′ (sense) and 5′-GTG TCA TAC GCT TTC TTT CCA T-3′ (antisense); FasL (278 bp), 5′-GGA TTG GGC CTG GGG ATG TTT CA-3′ (sense) and 5′-AGC CCA GTT TCA TTG ATC ACA AGG-3′ (antisense); *β*-actin (247 bp), 5′-CAA GAG ATG GCC ACG GCT GCT-3′ (sense) and 5′-TCC TTC TGC ATC CTG TCG GCA-3′ (antisense). PCR products were analyzed by 1.5% agarose gel electrophoresis and visualized with ethidium bromide. The intensity of the bands obtained from the RT-PCR product was estimated with a Scion Image Instrument (Scion Corp.; Frederick, MD, USA).

### 2.8. Statistical Analysis

Data are expressed as the mean  ±  S.E. for the number of experiments. Statistical analyses were conducted using Sigmaplot software (version 11.0). Comparisons between the two groups were analyzed using the Student's *t*-test. ∗*P* < 0.05, ∗∗*P* < 0.01, and ∗∗∗*P* < 0.001 were considered statistically significant. All figures shown represent results from at least three independent experiments with a similar pattern.

## 3. Results

### 3.1. Effect of TPL on Growth of Melanoma Cells

**Figure 1:**
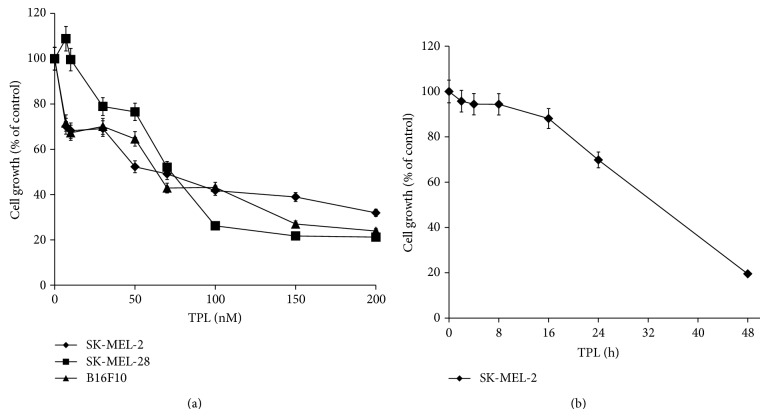
Effect of TPL on viability of melanoma cells. (a) Human melanoma cell lines, SK-MEL-2 and SK-MEL-28, and B16F10 mouse melanoma cells were treated with various concentrations of TPL for 24 h. (b) Time-dependent effect of TPL on SK-MEL-2 cell growth after treatment of 50 nM TPL. Cell growth was measured by MTT assay. Data were presented as percentage of control and were mean ± SEM (*n* = 3-4).

To investigate the effects of TPL on cell proliferation, melanoma cells were treated with TPL at different dose levels for 24 h and subjected to an MTT assay. As shown in Figure 1(a), TPL treatment for 24 h inhibited cell growth in a dose-dependent manner, with approximately 50% inhibition at 63.6, 70.5, and 89.6 nM on SK-MEL-2, SK-MEL-28 human melanoma cells, and B16F10 mouse melanoma cells, respectively. Treatment with 100 nM TPL resulted in about 60% to 80% reduction in growth of melanoma cells. We examined the time-dependent effect of TPL on growth inhibition in human SK-MEL-2 melanoma cells that showed inhibitory effect on cell growth at the lowest concentration of TPL among melanoma cell lines. Treatment with 50 nM TPL markedly inhibited cell growth at incubation time of more than 24 h (Figure 1(b)).

### 3.2. TPL Induces Apoptosis in SK-MEL-2 Melanoma Cells

**Figure 2:**
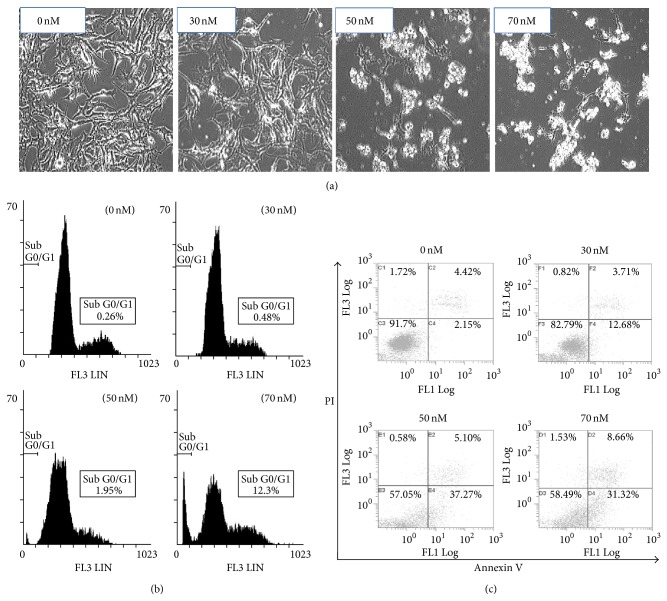
Effect of TPL on apoptosis of SK-MEL-2 cells. SK-MEL-2 cells were treated with 0, 30, 50, and 70 nM of TPL for 24 h. (a) Cells morphology was evaluated under the microscope. (b) Cells were washed, fixed, stained with propidium iodide (PI), and analyzed for DNA content by flow cytometry. (c) Apoptotic cells were measured by Annexin V assay and followed by analysis with flow cytometry.

To examine whether TPL inhibits the proliferation of melanoma cells by inducing apoptosis, the effects of TPL on the induction of apoptosis in SK-MEL-2 melanoma cells were examined. After treatment with different concentrations of TPL for 24 h, the cell morphology changed to irregular and shrunk shape with the increase in the TPL concentration (Figure 2(a)), suggesting that the cells were undergoing apoptosis by TPL. Furthermore, flow cytometric analysis also revealed that TPL treatment increased accumulation of cells at the apoptotic sub-G1 phase in a dose-dependent manner (Figure 2(b)). The number of cells at G2/M phase also increased in a dose-dependent manner (data not shown). To further confirm apoptosis of cells by TPL treatment, the percentage of apoptotic cells was determined by Annexin V-PI double staining after treatment with different concentrations of TPL for 24 h. Annexin V-FITC binds to exposed phosphatidylserine on apoptotic and necrotic cells, and PI staining exhibits entry into the late apoptotic cells and necrotic cells. The result showed the dose-dependent increase in SK-MEL-2 cells positive for Annexin V and Annexin V-PI (Figure 2(c)). These results indicate that the cytotoxic effect of TPL on SK-MEL-2 melanoma cells is associated with its apoptosis-inducing activity.

### 3.3. TPL Induces Apoptosis through Extrinsic and Intrinsic Pathways in SK-MEL-2 Melanoma Cells

**Figure 3:**
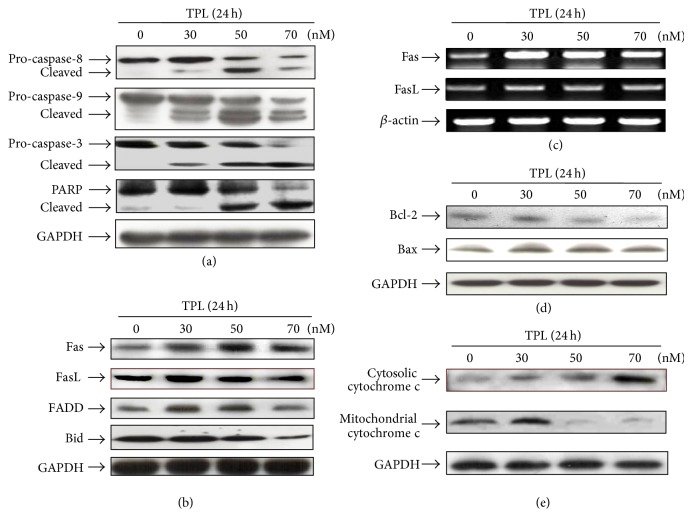
Effects of TPL on caspases activation in SK-MEL-2 cells. Human SK-MEL-2 cells were treated with 0, 30, 50, and 70 nM of TPL for 24 h, and then cells were collected. (a) Cell lysates were subjected to Western blotting analysis to detect caspase-8, -9, and -3 and PARP. (b and c) Effects of TPL on protein expressions related to apoptosis through Fas death receptor. (b) The cell lysates were subjected to Western blot analysis. Each protein was detected using Fas, FasL, FADD and Bid antibodies. (c) Total RNA was extracted in SK-MEL2 cells treated with TPL. The mRNA levels of Fas receptor and Fas ligand were examined by RT-PCR. (d and e) Effects of TPL on protein expressions related to apoptosis through mitochondria. (d) Each protein was detected using Bcl-2, Bax. (e) The cytosolic and mitochondrial fraction proteins were collected and then detected using cytochrome c antibody. GAPDH and *β*-actin were used as internal controls.

It is known that TPL induces cancer cell apoptosis directly via the mitochondrial (intrinsic) and/or the death receptor-mediated (extrinsic) pathways [9]. Caspase-8 and -9 play important roles in induction of apoptosis through intrinsic and extrinsic pathways, respectively [22]. To clarify the pathway by which TPL induces apoptosis in SK-MEL-2 cells, protein levels of caspase-8 and caspase-9 were analyzed by Western blot analysis after the cells were exposed to the indicated concentration of TPL for 24 h. As shown in Figure 3(a), protein levels of both pro-caspase-8 and pro-caspase-9 were decreased in dose-dependent manner, whereas those of their cleaved forms were increased, indicating activation of both caspases by TPL. Caspase-3 is activated through cleavage into two smaller subunits by caspase-8 or caspase-9 when the cells undergo apoptosis and cleaves a number of proteins that are essential for cell survival, such as PARP [22]. TPL also caused the activation of caspase-3 and subsequent cleavage of PARP. This result indicates that TPL-induced apoptosis in SK-MEL-2 cells was mediated by activations of caspase-3, -8, and -9, suggesting that TPL affects two pathways. Therefore, we checked downstream of extrinsic and intrinsic pathways in TPL-treated cells. In the extrinsic pathway, Fas aggregation is known to recruit FADD protein to the plasma membrane, which in turn activates pro-caspase-8 [9]. To verify TPL-mediated apoptosis through extrinsic pathway in SK-MEL-2 cells, levels of Fas, FasL, and FADD were investigated. As shown in Figures 3(b) and 3(c), the expression levels of Fas and FADD were increased in TPL-treated cells, whereas FasL showed no appreciable change in the expression levels of mRNA and protein. This result suggests that TPL-induced Fas aggregation is independent of its ligand. This result suggests that TPL-induced Fas aggregation is independent of its ligand. Moreover, the level of Bid was decreased, which presumably resulted in production of truncated Bid form (tBid). Bid is cleaved by active caspase-8, resulting in generating tBid, and translocates to the mitochondria and then enhances cytochrome c release by its interaction with Bax or Bak [22]. Thus, this result suggests that TPL-induced apoptosis in SK-MEL-2 cells can be mediated through intrinsic pathway via extrinsic pathway mediated by tBid. To confirm TPL-mediated apoptosis through intrinsic pathway in SK-MEL-2 cells, levels of Bcl-2, Bax, and cytochrome c were investigated. As shown in Figure 3(d), the level of Bcl-2, antiapoptotic protein, was decreased, whereas the level of Bax, proapoptotic protein, was increased after TPL exposure. Furthermore, cytochrome c was released from mitochondria to cytosol in a dose-dependent manner by TPL treatment (Figure 3(e)). This result indicates that TPL-induced apoptosis in SK-MEL-2 cells was mediated through intrinsic (mitochondrial-mediated) pathway.

**Figure 4:**
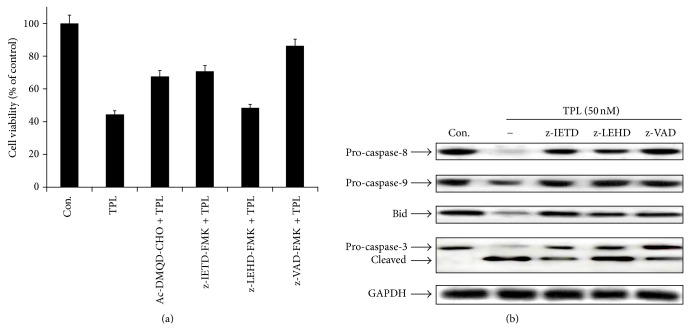
Effect of caspase inhibitors on TPL-induced apoptosis in SK-MEL-2 cells. Cells were incubated with 100 *μ*M of caspase inhibitors z-IETD-FMK (caspase-8 inhibitor), z-LEHD-FMK (caspase-9 inhibitor) or z-VAD-FMK (pan-caspase inhibitor) for 1 h and then coincubated with 50 nM of TPL for 24 h. (a) Cell viability was measured by MTT assay. Data were presented as percentage of control and were mean ± S.E. (*n* = 3). ∗∗*P* < 0.01 and ∗∗∗*P* < 0.001 versus TPL treatment. (b) Cell lysates were subjected to Western blotting analysis to detect caspase-8, -9, and -3 and Bid. GAPDH was used as internal control.

To get further insight into the mechanism of TPL-mediated apoptosis in SK-MEL-2 cells, we used a series of specific pharmacologic inhibitors of caspase activity (Figure 4(a)). Pretreatment of cells with the pan-caspase inhibitor Z-VAD-FMK significantly inhibited TPL-induced apoptosis, suggesting a requirement of caspase activity for TPL-induced apoptosis in SK-MEL-2 cells. Interestingly, pretreatment with caspase-9 inhibitor, Z-LEHD-FMK, had no significant effect, whereas caspase-3 inhibitor, AC-DMQD-CHO, and caspase-8 inhibitor, Z-IETD-FMK, significantly suppressed TPL-induced apoptosis. These results indicate that TPL-induced apoptosis was mainly mediated through the activations of caspase-3 and -8 in SK-MEL-2 cells and suggest that TPL can induce apoptosis through extrinsic pathway.

Based on the previous results, we identified the protein levels of caspases and Bid by treatment of caspase inhibitors. As shown in Figure 4(b), pretreatment of cells with the pan-caspase inhibitor Z-VAD-FMK and caspase-8 inhibitor Z-IETD-FMK significantly blocked caspase-3 cleavage by TPL, but caspase-9 inhibitor Z-LEHD-FMK had no significant effect, suggesting that caspase-3 activation is mainly mediated by caspase-8. In addition, the level of pro-caspase-9 reduced by TPL was not decreased by caspase-8 inhibitor Z-IETD-FMK, indicating that caspase-9 activation is mediated by active caspase-8. The level of Bid protein reduced by TPL was not also decreased by caspase-8 inhibitor Z-IETD-FMK, indicating that Bid cleavage to produce tBid is mediated by active caspase-8. Taken together, these results suggest that TPL-induced apoptosis in SK-MEL-2 cells can be mediated through the death receptor-mediated (extrinsic) and the mitochondrial (intrinsic) pathways.

### 3.4. TPL Induces FAK Dephosphorylation and Cleavage in SK-MEL-2 Cells

**Figure 5:**
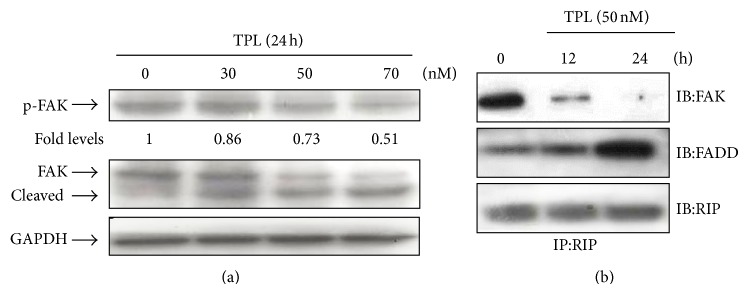
Effect of TPL on the level of FAK and FAK interaction with RIP in SK-MEL-2 cells. (a) Cells were treated with 0, 30, 50, and 70 nM of TPL, and then cells were collected. Cell lysates were analyzed by Western blotting. Each protein was detected using p-FAK, FAK antibodies. The relative abundance of each p-FAK band to its own FAK plus cleaved FAK was quantified by densitometry, and the control levels were set to 1. (b) Cells were either not treated or treated with TPL (50 nM) for 0, 12, and 24 h, and lysates were immunoprecipitated and immunoblotted as indicated.

Focal adhesion kinase (FAK) is generally overexpressed in cancer cells. FAK has been shown to be a key component in cell proliferation, survival regulation and especially protecting apoptosis [23]. FAK has been reported to be dephosphorylated at Tyr^397^ and cleaved during apoptosis, and FAK cleavage is mediated by caspases [24, 25]. Based on these reports, we investigated the modification of FAK in TPL-induced apoptosis. As shown in Figure 5(a), TPL treatment caused dephosphorylation of FAK at Tyr^397^ in dose-dependent manner, and FAK phosphorylation started to decrease at 50 nM TPL. In addition, FAK was also cleaved in dose-dependent manner in TPL-treated cells. It is known that FAK/receptor-interacting protein (RIP) complex disassociates upon dephosphorylation of FAK. Furthermore, RIP is associated with FAK-mediated survival or Fas/CD95, FADD-mediated cell death [25–27]. We examined RIP interaction between FAK and RIP state under TPL treatment condition by immunoprecipitation. As shown in Figure 5(b), RIP interacted with FAK in TPL-untreated control. However, RIP interaction with FAK was decreased in TPL-induced apoptosis, whereas its binding to FADD was increased. These results suggest that change of RIP assembly between FAK and FADD modulates TPL-mediated apoptosis through FAK dephosphorylation and increment of FADD expression.

### 3.5. TPL-Induced Apoptosis Occurs through Caspase-Mediated Cleavage of FAK

Recent studies demonstrated that FAK cleavage during induction of apoptosis is mediated by caspases [28–30]. Treatment of SK-MEL-2 melanoma cells with TPL at various doses for 24 h caused activations of caspase-8, -9, and -3 (Figure 3(a)) and TPL-induced FAK cleavage during apoptosis (Figure 5(a)).

**Figure 6:**
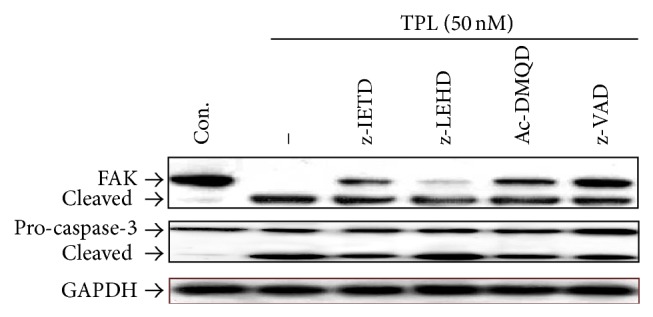
Effect of TPL on caspase-dependent cleavage of FAK in SK-MEL-2 cells. Cells were incubated with 100 *μ*M of caspase inhibitors, z-IETD-FMK, z-LEHD-FMK, Ac-DMQD-FMK, or z-VAD-FMK, for 1 h and then coincubated with 50 nM of TPL for 24 h, and then cells were collected. Cell lysates were analyzed by Western blotting. Each protein was detected using caspase-3 and FAK antibodies. GAPDH was used as an internal control.

To further examine whether caspase activation was related to cleavage of FAK in TPL-induced apoptosis, we checked the protein levels of FAK and caspase-3 by treatment of caspase inhibitors. As shown in Figure 6, pretreatment of cells with the pan-caspase inhibitor Z-VAD-FMK and caspase-8 inhibitor Z-IETD-FMK significantly blocked FAK and caspase-3 cleavages by TPL, but caspase-9 inhibitor Z-LEHD-FMK had no significant effect. This result indicates that FAK cleavage was induced by caspase-3 activated through caspase-8 activation of extrinsic pathway in TPL-treated cells.

## 4. Discussion

Although it was very recently reported that TPL inhibits the proliferation of human melanoma A375 cells and induces apoptosis by a caspase-dependent pathway [20], the precise mechanism of apoptosis induced by TPL in human melanoma cells is still unknown. In this study, we have demonstrated for the first time that TPL induces apoptosis through both mitochondrial (intrinsic) and the death receptor-mediated (extrinsic) pathways in human melanoma cells. Human melanoma cells are known to have the resistance to induction of apoptosis by conventional chemotherapeutic and biological agents [31]. Our result in this study revealed that human and mouse melanoma cells tested were all sensitive to the growth inhibitory effects of TPL, and its effect was correlated with apoptosis induction, as evidenced by flow cytometric analysis and Annexin V-PI double staining. We also found here that TPL induces activations of caspase-3, -8, and -9 and subsequent cleavage of PARP, which are regarded as hallmark of apoptosis. These results provide a reasonable explanation for induction of apoptosis by TPL in SK-MEL-2 cells.

TPL-induced apoptosis is known to be mediated through the mitochondrial (intrinsic) and/or the death receptor-mediated (extrinsic) pathways [9]. Activations of caspase-8 and -9 are characteristic of extrinsic and intrinsic pathways, respectively. The extrinsic pathway is initiated by the interaction of the death receptor (Fas/CD95) with its ligand (FasL). The Fas receptor activated by the binding of FasL to Fas recruits an adaptor molecule, FADD, and pro-caspase-8 into a death-inducing signaling complex (DISC), resulting in the activation of pro-caspase-8, and subsequently promotes the activation of downstream pro-caspase-3 [22]. Our result revealed that TPL increased significantly the levels of Fas and FADD in SK-MEL-2 cells, whereas FasL showed only a slight increase. This change in the ratio of Fas/FasL level was observed in the expression levels of mRNA and protein, indicating that TPL selectively induces Fas expression in human SK-MEL-2 cells. To the best of our knowledge, this is the first report showing that TPL upregulates Fas expression independently of its ligand in human melanoma cells. A similar mechanism of Fas ligand-independent, FADD-mediated activation of the Fas death pathway was observed in human melanoma cells treated with curcumin [32], human colon and leukemic cell lines treated with cisplatin and etoposide [33, 34], and head and neck squamous carcinoma cells treated with the synthetic retinoid MX3350-1 [35]. It has been reported that upregulation of Fas in response of anticancer drugs renders cancer cell sensitive to chemotherapy [36]. In light of these reports, our finding suggests that upregulation of Fas and constitutive FasL are important for the induction of apoptosis by TPL in SK-MEL-2 cells.

Our results presented here also demonstrated that TPL activates intrinsic pathway that is initiated with cytochrome c release from mitochondria and the activation of pro-caspase-9 cascade. It is known that Bcl-2 and its family proteins including tBid and Bax are important modulators of cytochrome c release from mitochondria [22, 37]. Bcl-2 is an antiapoptotic protein that regulates the mitochondrial release of cytochrome c, the interaction of Apaf-1 with pro-caspase-9, and binding to Bax, whereas Bax is a proapoptotic Bcl-2 homologue that exists in the cytoplasm or in the cell membrane and can antagonize the protective role of Bcl-2 [38]. We found in this study that TPL decreased Bcl-2 level and increased Bax level in SK-MEL-2 cell. Moreover, the increased cytochrome c level in the cytosol compared to mitochondria was observed in TPL-treated cells. These results clearly indicate that the increased Bax/Bcl-2 ratio contributed to the disruption of mitochondrial potential and the release of cytochrome c into cytosol, which resulted in activation of pro-caspase-9. Bid is a proapoptotic protein that connects extrinsic pathway with intrinsic pathway and tBid produced by active caspase-8 translocates to the mitochondria and then enhances cytochrome c release by its interaction with Bax [37, 38]. Our findings also demonstrated that Bid mediates the communication between extrinsic pathway and intrinsic pathway, as evidenced by cell viability and Western blot analysis using a series of specific caspase inhibitors.

It is known that FAK, a nonreceptor tyrosine kinase, regulates cancer cell survival and death receptor-mediated cell death pathway [39], and inhibition of FAK activationcauses cell rounding, loss of adhesion, and apoptosis in human cancer cell lines [40]. It was reported that phosphorylation of Tyr^397^ of FAK is crucial for FAK activation, and its dephosphorylation triggers cell detachment by disassembly of focal adhesion, resulting in apoptotic cell death or anoikis [41]. FAK is also known to be proteolytically cleaved by caspases during induction of apoptosis. Several studies have shown that FAK cleavage is mediated by caspase-3 or caspase-8 [28–30]. Previous study showed that Geraniin-induced apoptosis occurs by caspase-3-mediated FAK cleavage through upregulation of Fas ligand expression in human melanoma cells [42]. Recent study also revealed that TPL induced FAK cleavage in MCF-7 breast cancer cells [43]. In this study, we also demonstrated that TPL inhibits phosphorylation of Tyr^397^ of FAK and induces cleavage of FAK by caspase-3 activated through caspase-8 activation of extrinsic pathway in SK-MEL-2 cells, as evidenced by Western blot analysis using caspase inhibitors. Taken together, the present results suggest that TPL-induced apoptosis in SK-MEL-2 cells is triggered through dephosphorylation of FAK and its cleavage by caspase-8-mediated activation of caspase-3 via upregulation of Fas expression of extrinsic pathway.

It was previously reported that receptor-interacting protein (RIP) interacts with FAK to suppress apoptosis [44] and takes part in both cell survival and apoptotic cell death [25]. Moreover, recent study has shown that RIP shuttles between CD95/Fas and FAK to mediate anoikis induced by the Fas/CD95 death receptor [25]. Based on these reports, the interaction of RIP with FAK in TPL-treated SK-MEL-2 cells was investigated by immunoprecipitation using RIP-specific antibody. In this study, we firstly demonstrated that interaction of RIP with FAK decreased in time-dependent manner in TPL-treated human melanoma cells, whereas that of RIP with Fas/FADD complex increased, suggesting that apoptotic condition induced by TPL triggers the dissociation of RIP from FAK through FAK dephosphorylation and enhances the formation of RIP/Fas complex formation initiating cell death via increase of Fas and FADD expression levels.

## 5. Conclusion

**Figure 7:**
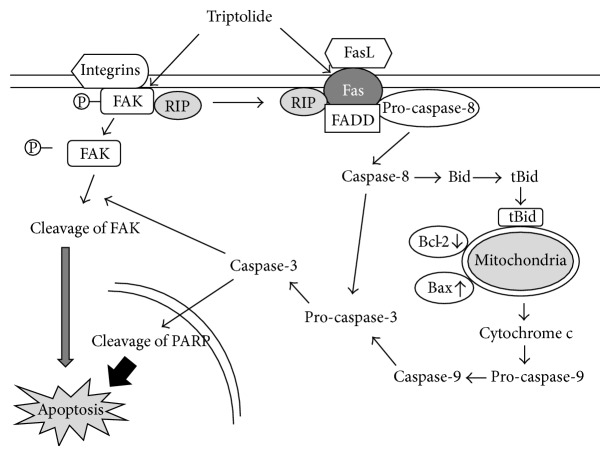
Schematic illustration of mechanism underlying TPL-induced apoptosis in SK-MEL-2 cells.

As illustrated in Figure 7, our data showed that TPL induced apoptosis in human melanoma cells through the death receptor-mediated (extrinsic) and the mitochondrial (intrinsic) pathways. In the death receptor-mediated (extrinsic) pathway, TPL can induce directly the activation of pro-caspase-8 through upregulation of Fas expression, which subsequently promotes proteolytic processing of pro-caspase-3 and Bid. Alternatively, in the mitochondrial (intrinsic) pathways, the formation of tBid mediated by active caspase-8 induces indirectly the increased Bax/Bcl-2 ratio, resulting in the disruption of mitochondrial potential and the release of cytochrome c into cytosol, which subsequently causes activation of pro-caspase-9 followed by caspase-3 activation. The activated caspase-3 leads to cleavage of PARP, resulting in induction of TPL-induced apoptosis in human SK-MEL-2 melanoma cells. Also, TPL can lead to apoptosis in SK-MEL-2 cells through dephosphorylation of FAK and its cleavage by caspase-8-mediated caspase-3 activation via upregulation of Fas expression of extrinsic pathway. Although more works are required to further elucidate the FAK signaling pathway leading to the association of RIP and FAK in TPL-induced apoptosis, our data presented here may provide more useful information for understanding the molecular mechanisms of TPL-induced apoptosis in human melanoma cells.
